# The fungal stronghold: biofilms in hemodialysis catheters, diagnostic pitfalls, and the challenge of catheter salvage

**DOI:** 10.3389/fcimb.2026.1774864

**Published:** 2026-03-18

**Authors:** Jie Shi, Naiying Lan, Fanzhou Zeng, Nanmei Liu, Cheng Xue, Bo Yang

**Affiliations:** 1 Department of Disease Control and Prevention, Naval Medical Center of People's Liberation Army (PLA), Naval Medical University, Shanghai, China; 2 Department of Nephrology, Naval Medical Center of People's Liberation Army (PLA), Naval Medical University, Shanghai, China; 3 Department of Cellular Biology and Anatomy, Medical College of Georgia, Augusta University, Augusta, GA, United States; 4 Department of Nephrology, Shanghai Changzheng Hospital, Second Affiliated Hospital of Naval Medical University, Shanghai, China

**Keywords:** antifungal lock therapy, catheter salvage, catheter-related bloodstream infection, fungal biofilm, hemodialysis

## Abstract

The management of fungal catheter-related bloodstream infections (CRBSIs) in the hemodialysis population represents a critical collision between rigorous infectious disease guidelines and the grim clinical reality of vascular access exhaustion. While guidelines from the IDSA and KDIGO unequivocally recommend immediate catheter removal to prevent metastatic complications, nephrologists are frequently confronted with patients for whom the current catheter represents the last viable lifeline. This review provides a comprehensive analysis of the “catheter salvage” dilemma, moving beyond superficial treatment algorithms to explore the molecular and structural mechanisms that make fungal biofilms a formidable adversary. We dissect the pathogenesis of *Candida* colonization on abiotic surfaces (silicone and polyurethane), detailing the transition from yeast to hyphal structures and the secretion of a complex extracellular matrix (ECM). We highlight how the ECM, rich in β-1,3 glucan, acts as a physical shield that sequesters azoles, rendering standard systemic therapy ineffective despite *in vitro* susceptibility. Furthermore, we discuss the role of metabolically dormant “persister cells” in driving high relapse rates and analyze the epidemiological shift toward *Candida parapsilosis*, a pathogen with a unique affinity for foreign bodies and parenteral nutrition lines. Diagnostically, we scrutinize the limitations of traditional blood cultures and the “Differential Time to Positivity” (DTP) criteria, arguing that the slower growth kinetics of fungi render DTP unreliable compared to bacterial infections. The core of this review evaluates the efficacy and safety of Antifungal Lock Therapy (ALT) as a bridging strategy. We provide a comparative analysis of lock agents, contrasting the broad lytic potential of ethanol (the “nuclear option”) with the safety profile of taurolidine and the pharmacological nuances of amphotericin B and echinocandins. Ultimately, we propose that while catheter removal remains the gold standard, a nuanced mastery of biofilm biology and lock therapy protocols is essential for managing complex, access-challenged patients where immediate removal is not feasible.

## 1 Introduction

The central venous catheter (CVC) occupies a paradoxical space in modern nephrology: it is simultaneously the hemodialysis patient’s essential lifeline and their greatest vulnerability. For the vast majority of patients with End-Stage Kidney Disease (ESKD), the arteriovenous fistula remains the vascular access of choice; (Lok et al., 2025) however, the CVC remains a ubiquitous reality, often serving as the bridge to permanent access or, increasingly, as the destination therapy for those with “exhausted access.” (Viecelli and Lok, 2019; Pride et al., 2024) It is within this context that Catheter-Related Bloodstream Infections (CRBSIs) emerge as a leading cause of morbidity and mortality (Almeida et al., 2022). Among these, fungal infections—predominantly caused by Candida species—carry a particularly grave prognosis (Kumar and Snitowsky, 2024), with mortality rates often exceeding those of bacterial sepsis (with fungal CRBSI mortality often over 40%, compared to 12% to 20% for typical bacterial CRBSIs) (Almirante et al., 2005; Landry and Braden, 2014; Yu et al., 2025) due to the high risk of metastatic seeding to the eye, heart valves, and bone.

The medical consensus regarding fungal CRBSI is rigid and well-founded. The Infectious Diseases Society of America (IDSA), in their 2016 guidelines (Pappas et al., 2016), along with the Kidney Disease: Improving Global Outcomes (KDIGO) workgroups, mandates a clear standard of care: the identification of Candida species from a catheter or peripheral blood culture requires the immediate removal of the indwelling device. This recommendation is underpinned by substantial evidence demonstrating that catheter retention is an independent predictor of persistent fungemia, treatment failure, and increased mortality (Wilcox, 2009; Damonti et al., 2017). Unlike coagulase-negative staphylococci, where catheter salvage is a recognized and frequently successful strategy, the presence of fungi has historically been viewed as an absolute contraindication to catheter retention.

However, this binary decision framework—infection equals removal—disintegrates when applied to the growing demographic of the “access-exhausted” patient (Pride et al., 2024). Nephrologists frequently manage patients who have survived decades of renal replacement therapy, enduring the failure of multiple arteriovenous fistulas and grafts, the occlusion of central veins, and the loss of peritoneal dialysis viability due to encapsulating peritoneal sclerosis or a “hostile abdomen.” For such a patient, the infected tunneled catheter in the right internal jugular vein or a translumbar position may represent the absolute limit of vascular access. Removing such a catheter is not merely a procedural inconvenience; it is an event that precipitates significant clinical morbidity and may necessitate a transition to conservative kidney management.

Consequently, clinicians are forced into a “Clinical Dilemma” where strict adherence to guidelines may precipitate an immediate survival crisis. In these scenarios, “catheter salvage” is not a refusal to treat, but a calculated, high-stakes risk taken to preserve the patient’s immediate ability to receive dialysis. The failure of systemic antifungal therapy to sterilize retained catheters is directly linked to the unique recalcitrance of the biofilm phenotype rather than classical genetic antimicrobial resistance (Wang et al., 2024; Ramage et al., 2025). This biological fortress renders standard susceptibility testing irrelevant, as a Candida isolate that appears susceptible to fluconazole in a planktonic state may be completely impervious to the drug when encased in the catheter’s extracellular matrix.

This review justifies its existence by addressing this precise gap between the guideline-mandated ideal and the complex clinical reality. We aim to provide a comprehensive, mechanistic understanding of why fungal biofilms are so difficult to eradicate and to rigorously evaluate the evidence for localized salvage strategies—specifically Antifungal Lock Therapy (ALT). By understanding the “enemy within” the catheter lumen, clinicians can make more informed, risk-stratified decisions when backed into the corner of access exhaustion, using advanced pharmacological strategies to attempt salvage as a bridge to stability.

## 2 Pathogenesis: the “enemy within” (biofilm mechanics)

**Figure 1:**
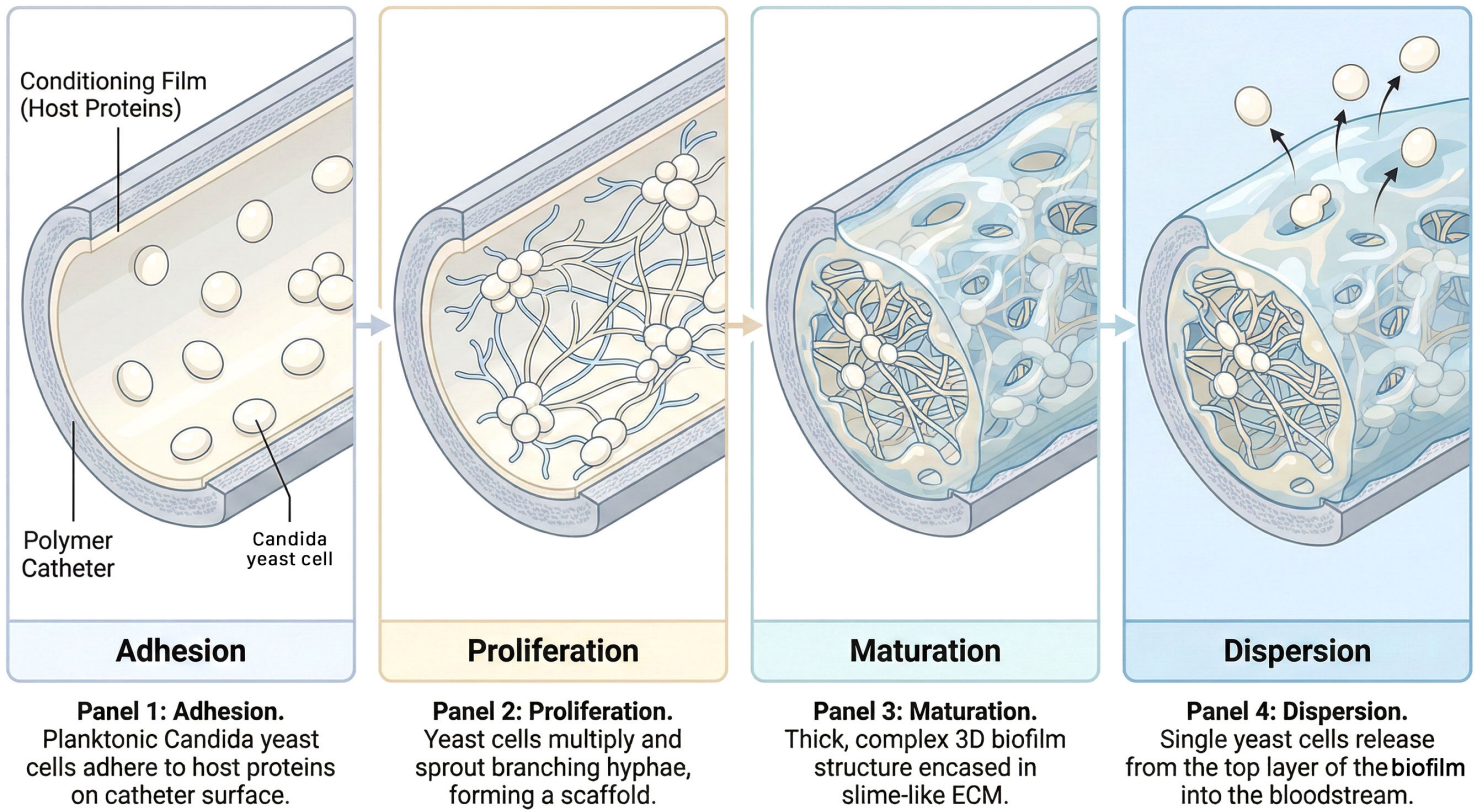
The lifecycle of a fungal biofilm on a hemodialysis catheter. This schematic illustrates the sequential development of a Candida biofilm on a polymer surface. (Panel 1) Adhesion: Planktonic yeast cells adhere to the “conditioning film” of host proteins (fibrinogen, fibronectin) that coats the catheter surface minutes after insertion. (Panel 2) Proliferation: Yeast cells replicate and undergo a morphological switch, sprouting branching hyphae that form the architectural scaffold of the biofilm. (Panel 3) Maturation: The fungal community becomes encased in a thick Extracellular Matrix (ECM), a slime-like protective barrier composed of polysaccharides and proteins. (Panel 4) Dispersion: Specialized “pioneer” yeast cells are released from the upper layer of the biofilm into the bloodstream, serving as a source for metastatic infection and intermittent fungemia.

To successfully manage or salvage a fungal CRBSI, one must first understand the fundamental biology of the adversary. A fungal biofilm is not merely an aggregation of cells; it is a structured, metabolically cooperative community that exhibits a distinct developmental program (Kernien et al., 2018). This transformation from a free-floating yeast cell to a sessile, biofilm-embedded community fundamentally alters the organism’s interaction with antifungal agents and the host immune system. (**Figure 1**).

### 2.1 The developmental cascade: adhesion to dispersion

The genesis of a *Candida* biofilm on a hemodialysis catheter follows a sequential, highly regulated lifecycle that transforms the device surface into a fungal stronghold.

The initial phase is adhesion, occurring within minutes to hours of catheter exposure. Planktonic yeast cells suspended in the bloodstream adhere to the catheter surface. While early *in vitro* studies focused on the interaction between fungal cells and bare polymer surfaces, the clinical reality is that strictly “abiotic” adhesion is rare. Immediately upon insertion, a catheter is coated in a “conditioning film” of host plasma proteins, including fibrinogen, fibronectin, albumin, and laminin. *Candida* species possess a specialized arsenal of cell wall proteins known as adhesins, most notably the Agglutinin-Like Sequence (ALS) family (e.g., Als3) and Hwp1, which bind specifically to these host proteins (Chandra and Mukherjee, 2015; Cavalheiro and Teixeira, 2018; Branco et al., 2023). This transforms the catheter from a foreign object into a biological substrate that the fungus recognizes as a site for colonization. The surface properties of the catheter material, such as hydrophobicity and roughness, play a modulatory role in this initial attachment, with hydrophobic surfaces generally favoring adhesion (Gulati et al., 2018).

Following attachment, the cells enter the Proliferation phase. The anchored yeast cells replicate to form a basal layer of microcolonies. This stage is characterized by rapid metabolic activity and the initial production of extracellular polymeric substances (Akbari and Kjellerup, 2015).

The critical turning point in biofilm pathogenicity is the maturation phase. For *C. albicans*, this involves a profound morphological transition. Yeast cells differentiate into elongated hyphae and pseudohyphae. This yeast-to-hyphae transition is not merely a structural change; it provides the architectural scaffold for the biofilm. The long hyphal elements intertwine, stabilizing the three-dimensional structure and allowing the development of water channels that facilitate the diffusion of nutrients and oxygen deep into the biomass (Boyce and Andrianopoulos, 2015; Wijaya et al., 2023). It is during this phase that the cells actively secrete the Extracellular Matrix (ECM), a complex polymeric substance that encases the cells and cements the structure.

The final stage of the lifecycle is dispersion. The mature biofilm is not a static entity; it is a reservoir for dissemination. Non-adherent yeast cells are released from the upper layers of the biofilm back into the bloodstream to seed other organs or colonize new sites. This active detachment process is regulated by specific genetic programs, such as the transcriptional regulators Nrg1 and Pes1, and is responsible for the clinical presentation of intermittent candidemia (Shen et al., 2008; Koch et al., 2018). A patient may have negative blood cultures one day and positive the next, as the biofilm periodically sheds “pioneer” cells. This cycle of shedding renders single negative blood cultures unreliable as proof of cure and underscores the danger of “silent colonization”.

### 2.2 The role of the extracellular matrix

The Extracellular Matrix (ECM) is the defining feature of the biofilm phenotype and the primary mechanism of drug resistance. Unlike bacterial biofilms, which are often dominated by polysaccharides (Balducci et al., 2023; Zhao et al., 2023), the fungal ECM is a complex amalgam of polysaccharides (specifically beta-1,3 glucan and mannan), proteins, lipids, and extracellular DNA (eDNA) (Chandra and Mukherjee, 2015; Perlin, 2015).

The ECM functions as a sophisticated physical shield and a drug sequestration sink. Research has definitively demonstrated that beta-1,3 glucans in the matrix bind avidly to common antifungal agents, particularly azoles (like fluconazole) and polyenes (like amphotericin B) (Mitchell et al., 2013). This sequestration effect prevents the drug from reaching the fungal cell membrane in lethal concentrations. Consequently, the Minimum Inhibitory Concentration (MIC) for sessile biofilm cells can be up to 1,000 times higher than that for planktonic cells of the same strain. This phenomenon explains the “clinical failure” of fluconazole in catheter salvage: even if the strain is tested as “susceptible” in the lab, the drug is physically prevented from engaging the target within the biofilm. Interestingly, this sequestration is less pronounced with echinocandins, which target the synthesis of the matrix components themselves, a pharmacological distinction that dictates therapeutic choice (Chatzimoschou et al., 2011; Szymański et al., 2022).

### 2.3 Persister cells: the architects of relapse

Even if an antifungal agent successfully penetrates the ECM, it faces the challenge of “persister cells.” These are a small subpopulation of phenotypic variants within the biofilm that exist in a dormant, metabolically quiescent state (Wuyts et al., 2018). Unlike resistant mutants, persister cells do not possess genetic modifications; rather, they have entered a state of suspended animation. Because most antifungal agents—and antibiotics—target active cellular processes such as cell wall synthesis or sterol metabolism, they are largely ineffective against non-dividing cells (Desai et al., 2014; Atriwal et al., 2021).

Persister cells are the ultimate survival mechanism. They withstand the antifungal onslaught that kills the metabolically active majority of the biofilm. Once the therapy is discontinued, these cells revert to a metabolically active state and repopulate the biofilm, leading to a recurrence of infection (Sychev et al., 2009). This mechanism is the primary driver of the high relapse rates observed in patients where catheter salvage is attempted without the use of high-concentration, lytic lock therapies capable of destroying dormant cells. The presence of persister cells necessitates prolonged therapy durations and the use of agents with broad lytic activity (Galdiero et al., 2020), such as ethanol or amphotericin B, to achieve sterilization.

### 2.4 Material science: silicone vs. polyurethane

The interaction between the fungal pathogen and the catheter material itself has been a subject of significant material science research. Historically, *in vitro* studies suggested that *C. albicans* formed more substantial, thicker biofilms on silicone elastomers and latex compared to polyurethane or polyvinyl chloride (PVC) (Hawser and Douglas, 1994; Chandra and Mukherjee, 2015). The inherent hydrophobicity of silicone was thought to facilitate the initial adhesion of hydrophobic yeast cells, promoting a more robust initial colonization.

However, more recent *in vivo* models and clinical analyses have added nuance to this view. Once a catheter is inserted into the bloodstream, the rapid deposition of the host conditioning film essentially masks the underlying physicochemical properties of the polymer. The fungus interacts primarily with the host proteins—fibrinogen and fibronectin—rather than the raw silicone or polyurethane (Andersen et al., 2022). Consequently, in the clinical setting, no standard catheter material confers immunity to fungal colonization. Both silicone and polyurethane catheters are highly vulnerable once the conditioning film is established. This has spurred research into novel material modifications, such as coating catheters with chitosan hydrogels or auranofin (a gold-containing compound), which have shown promise in reducing biofilm mass in preclinical models by inhibiting fungal metabolic activity and adhesion (Felix et al., 2023). Until such technologies become standard, however, the material of the catheter offers little protection against the establishment of the fungal stronghold.

### 2.5 Clinical implications summary

In summary, the clinical failure of standard systemic therapies against catheter-associated fungal infections is primarily driven by two factors: the Extracellular Matrix (ECM), which physically sequesters antifungal agents like azoles, and the presence of dormant persister cells, which survive the initial antifungal onslaught to cause relapse once therapy is withdrawn.

## 3 Epidemiology & the shift in pathogens

The epidemiology of fungal CRBSIs in hemodialysis units is undergoing a distinct and concerning shift. While *Candida albicans* remains the predominant pathogen in many settings, there is a rising prevalence of Non-albicans Candida (NAC) species, including *C. parapsilosis*, *C. glabrata*, and *C. tropicalis* (Pyrgos et al., 2009; Malinovská et al., 2023). This epidemiological shift toward NAC species has been observed globally over the past decade. It is important to note that while this is a worldwide trend, specific outbreaks are often dialysis-unit specific, largely driven by nosocomial transmission (Parslow and Thornton, 2022; Zoi et al., 2025).

### 3.1 The rise of *Candida parapsilosis*

*Candida parapsilosis* deserves specific attention in the context of catheter-related infections. Unlike *C. albicans*, which is typically an endogenous pathogen translocating from the patient’s own gastrointestinal tract (Singh et al., 2020), *C. parapsilosis* is frequently a commensal of the skin and is easily transmitted via the hands of healthcare workers. This makes it a quintessential nosocomial pathogen, often responsible for outbreaks in dialysis units and intensive care settings (Trofa et al., 2008).

Crucially, *C. parapsilosis* exhibits a unique biological affinity for foreign bodies. It is a potent biofilm former, although its biofilms differ structurally from those of *C. albicans*. *C. parapsilosis* biofilms are often thinner and less complex, lacking the dense hyphal architecture of *C. albicans*, but they are comprised of cells with high metabolic activity and strong adherence properties (Kuhn et al., 2002; Silva et al., 2009). Genetic analysis reveals that *C. parapsilosis* possesses distinct adhesin genes and regulatory networks—such as BCR1-independent pathways in some high-biofilm forming strains—that facilitate rapid colonization of parenteral nutrition lines and hemodialysis catheters (Pannanusorn et al., 2014). The organism’s ability to thrive in high-glucose environments, such as Total Parenteral Nutrition (TPN) admixtures, often mirrors its success in the protein-rich environment of a conditioning film, making it a formidable adversary in patients receiving intradialytic parenteral nutrition.

### 3.2 *Candida glabrata* and the resistance challenge

*Candida glabrata* presents a different but equally difficult challenge. Historically considered a non-pathogenic commensal, it has emerged as a significant cause of CRBSI, particularly in elderly and immunosuppressed populations. *C. glabrata* is generally a poorer biofilm former compared to *C. albicans* or *C. parapsilosis*, often forming monolayers rather than complex multilayered structures (Kuhn et al., 2002; Silva et al., 2009; Gonçalves et al., 2020). However, its danger lies in its resistance profile. *C. glabrata* is intrinsically less susceptible to azoles like fluconazole and can rapidly acquire resistance to echinocandins through mutations in the FKS genes (Alexander et al., 2013; Lotfali et al., 2021; Salazar et al., 2022). A biofilm of *C. glabrata*, even if structurally meager, can be incredibly difficult to eradicate due to this high baseline drug resistance, necessitating the use of high-dose echinocandins or lipid formulations of amphotericin B (Kucharíková et al., 2010; Rodrigues et al., 2016).

## 4 Diagnostic challenges

The diagnosis of fungal CRBSI is fraught with pitfalls that often lead to delayed treatment or missed diagnoses. To optimize clinical management, it is critical to explicitly distinguish between diagnostic tools used for systemic detection and those used for catheter source attribution. (**Table 1**).

**Table 1:** Comparison of bacterial vs. fungal CRBSI diagnostics.

Diagnostic modality	Bacterial CRBSI	Fungal CRBSI	Clinical implication
Blood Culture Sensitivity	High	Low (21-71%)	Negative culture does not rule out fungal biofilm.
Differential Time to Positivity (DTP)	Highly reliable for source attribution (>120 min)	Unreliable due to slow yeast growth kinetics	DTP should not be used to rule out fungal CRBSI.
T2Candida Panel	N/A	High sensitivity, rapid detection (3–5 hrs)	Excellent for systemic detection; does not confirm source.
Biomarkers	Procalcitonin (PCT)	Beta-D-Glucan (BDG)	BDG is adjunctive; cannot replace clinical judgment for source control.

CRBSI: Catheter-Related Bloodstream Infection.

### 4.1 Systemic detection: the sensitivity gap and “silent colonization”

Peripheral blood cultures have notoriously low sensitivity for candidemia, estimated at only 21-71% in some studies (Nguyen et al., 2012; Barantsevich and Barantsevich, 2022). In the context of a catheter-based biofilm, the shedding of fungal cells into the bloodstream is intermittent and often low-level. A negative peripheral culture does not rule out a colonized catheter. The biofilm can exist in a localized state, seeding the blood only sporadically or at levels below the detection threshold of standard automated blood culture systems (1–10 CFU/mL) (Brecher et al., 2001). This “silent colonization” is dangerous, as it can serve as a persistent reservoir for metastatic infection even when the patient appears clinically improved.

### 4.2 Tools for source attribution: the fallacy of differential time to positivity

For bacterial infections, Differential Time to Positivity (DTP) is a valuable diagnostic tool used to implicate the catheter as the source of infection without removing it (Dhaliwal and Daneman, 2023). If blood drawn from the CVC hub becomes positive more than 2 hours (120 minutes) earlier than blood drawn from a peripheral vein, it strongly suggests the catheter is the source, based on the assumption of a higher microbial load in the catheter lumen.

However, literature critically evaluates and often advises against the application of DTP to fungal infections. Fungal growth rates are significantly slower than bacteria. *Candida* species often require 24 to 48 hours or more to flag positive in automated systems, compared to 10–12 hours for Staphylococcus aureus (Maquelin et al., 2002). The replication kinetics of yeast do not align with the models built for bacteria. Studies have shown that DTP criteria established for bacteria generally perform poorly for *Candida*, with lower sensitivity and concordance (Mermel et al., 2009; Dhaliwal and Daneman, 2023). A negative or inconclusive DTP result should never be used to rule out a fungal CRBSI or to justify retaining a suspected catheter. The physics of yeast replication within the culture bottle simply render this tool unreliable for fungi.

### 4.3 Emerging non-culture diagnostics for systemic detection

Given these limitations, non-culture diagnostic methods are gaining traction, offering new avenues for detection and monitoring.

T2Candida: This magnetic resonance-based assay detects *Candida* species directly in whole blood without the need for culture. It works by detecting the agglomeration of superparamagnetic particles induced by the presence of amplified DNA. It offers a rapid turnaround time (3–5 hours) and significantly higher sensitivity than blood culture (over 90%), capable of detecting as few as 1–3 CFU/mL (Clancy et al., 2018; Arendrup et al., 2019). In the context of “salvage,” a T2Candida result could theoretically help monitor the clearance of fungemia more accurately than cultures, although its high cost and inability to provide susceptibility data are limiting factors. It is particularly useful for detecting the five most common species: *C. albicans*, *C. parapsilosis*, *C. tropicalis*, *C. krusei*, and *C. glabrata* (Shenoy and Joshi, 2022).

Beta-D-Glucan (BDG): This test detects (1,3)-beta-D-glucan, a cell wall component found in most fungi (except Zygomycetes and Cryptococcus). It has a high negative predictive value, making it useful for ruling out invasive fungal infection (Kazancioglu et al., 2022; Ullah et al., 2025). However, its utility in diagnosing catheter biofilm specifically is limited by potential false positives. Historically, cellulose-based hemodialysis membranes caused false-positive BDG results (Prattes et al., 2015; Cabanilla et al., 2023), although modern synthetic polysulfone membranes are less prone to this issue. A positive BDG indicates the presence of fungus but lacks specificity regarding the site of infection (e.g., catheter vs. gut translocation). Therefore, while useful as an adjunctive marker, it cannot replace clinical judgment in the diagnosis of CRBSI.

## 5 Management: the “pull vs. salvage” debate

The management of fungal CRBSI is the crucible where guidelines meet clinical necessity. The IDSA and KDIGO guidelines are clear and unequivocal: the catheter should be removed. This recommendation is based on robust data showing higher mortality, prolonged fungemia, and higher relapse rates when catheter retention is attempted (Lata et al., 2016; Rosenblatt et al., 2017). However, for the patient with “exhausted access,” removal is an event that precipitates significant clinical morbidity and may necessitate a transition to conservative kidney management. Thus, the “Salvage” approach, while controversial, must be mastered for these specific cases as a bridge to survival.

### 5.1 Systemic therapy: pharmacological realities

Systemic antifungal therapy is non-negotiable, whether the catheter is removed or retained. The choice of agent, however, is critical when biofilm is involved.

Azoles (Fluconazole): While fluconazole is the workhorse of antifungal therapy due to its oral availability and low cost, it is structurally ill-suited for the primary treatment of biofilm-associated infections. As discussed in the pathogenesis section, the ECM sequesters azoles, and efflux pumps (CDR1, CDR2, MDR1) in biofilm cells actively export the drug (Fanning and Mitchell, 2012; Li et al., 2025). Furthermore, fluconazole is largely fungistatic against *Candida*, meaning it inhibits growth but does not kill the organism. In the context of a foreign body infection where host defenses are impaired, fungistatic activity is often insufficient to sterilize the device. Fluconazole is best reserved for step-down therapy after the catheter has been removed or initially stabilized with more potent agents (Ruhnke, 2014).

Echinocandins (Micafungin/Caspofungin/Anidulafungin): These agents have emerged as the preferred first-line therapy for candidemia and are pharmacologically superior for biofilm-associated infections. They act by non-competitively inhibiting the synthesis of beta-1,3 glucan, a key component of both the fungal cell wall and the biofilm matrix (Bossche, 2002). By targeting the matrix structure itself, echinocandins can penetrate the biofilm more effectively than azoles. Furthermore, they are fungicidal against *Candida* species. Studies have demonstrated that echinocandins exhibit a “paradoxical” effect where they remain active against biofilms even at high concentrations, unlike azoles which lose efficacy due to sequestration (Katragkou et al., 2015; Swaminathan et al., 2018). For any attempt at catheter salvage, an echinocandin-based systemic regimen is mandatory to lower the fungal burden and prevent metastatic seeding.

### 5.2 Antifungal lock therapy for salvage

**Figure 2:**
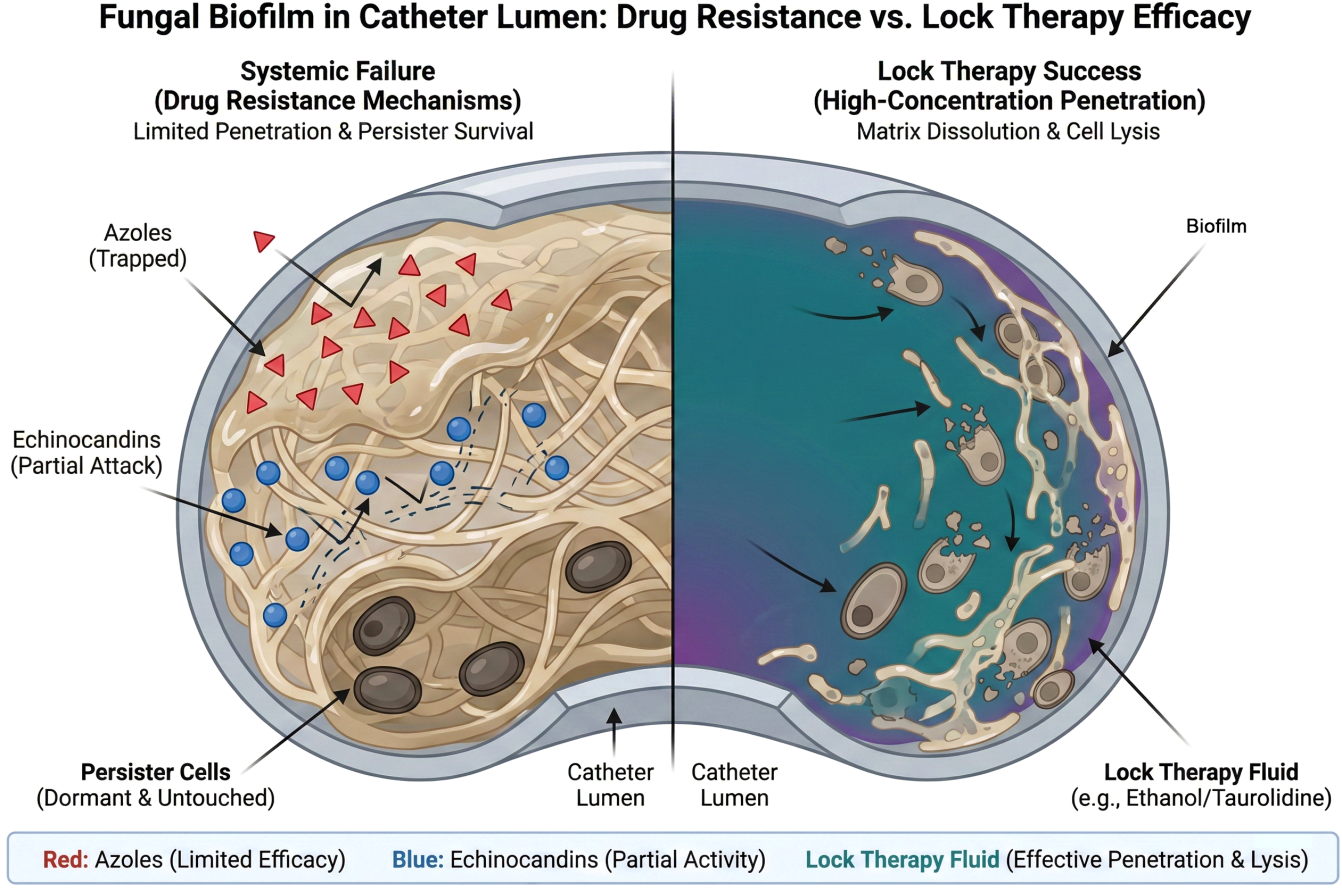
Mechanisms of systemic treatment failure vs. lock therapy success. A comparison of therapeutic efficacy within the infected catheter lumen. Left (Systemic Failure): Despite systemic administration, antifungal agents fail to sterilize the catheter. Azoles (red triangles) are physically sequestered by the Extracellular Matrix (ECM) and cannot reach the fungal cells. Echinocandins (blue circles) show partial activity but may not fully penetrate deep layers or eradicate dormant Persister Cells, which survive to cause relapse once therapy ceases. Right (Lock Therapy Success): High-concentration Lock Therapy fluid (e.g., Ethanol or Amphotericin B) saturates the lumen, successfully penetrating the ECM, lysing metabolically active cells, and eradicating the persister reservoir, resulting in catheter sterilization.

Systemic therapy alone cannot sterilize a colonized catheter because it cannot achieve sufficient concentrations within the lumen to overcome the “biofilm resistance factor” (which can be 1000x the planktonic MIC) (Seneviratne et al., 2008). This is where Antifungal Lock Therapy (ALT) becomes the linchpin of the salvage strategy. ALT involves instilling a high-concentration antimicrobial solution into the catheter lumen during the inter-dialytic period to dwell until the next session. (**Table 2**) (**Figure 2**).

**Table 2:** Antifungal catheter lock solutions: agents, compatibility, and practical considerations.

Agent	Concentration	Diluent	Anticoagulant compatibility	Spectrum & mechanism	Comments & caveats
Amphotericin B (Deoxycholate)	2.0 – 2.5 mg/mL	D5W (5% Dextrose)	Incompatible with saline. Limited *in vitro* data suggest possible short−term compatibility with heparin (≈5,000 U/mL), but clinical compatibility remains uncertain	Broad spectrum. Pore-forming fungicidal.	Most frequently reported antifungal lock solutions in case series. Protect from light and prepare aseptically to avoid precipitation. Typical dwell times 12–24 h or interdialytic. Concomitant use with heparin should be approached cautiously
Liposomal Amphotericin B	2.0 – 5.0 mg/mL	D5W (5% Dextrose)	Better compatibility profile than deoxycholate, but still requires care.	Broad spectrum; liposomal delivery may improve tissue and biofilm exposure in experimental models	Used mainly in refractory cases or when intolerance to conventional amphotericin B exists. Significantly higher cost. Clinical superiority over deoxycholate lock solutions has not been established
Ethanol	70% (v/v)	Sterile Water	Incompatible with Heparin (precipitates). Do not mix.	Ultra-broadest spectrum (Bacteria + Fungi). Lyses cells/denatures proteins.	Considered a last−resort catheter salvage strategy. Effective against mature biofilm but associated with catheter material degradation (especially polyurethane), thrombosis risk, and patient discomfort. Dwell time should be limited and catheter integrity monitored
Taurolidine-Citrate	1.35% - 2.0% Taurolidine + 4% Citrate	N/A (Pre-formulated)	Compatible (often pre-mixed).	Broad antimicrobial activity mainly for prevention; chemical modification of microbial cell walls	Strongest evidence supports prophylactic use rather than eradication of established high−burden fungal biofilm. Excellent safety profile and no documented resistance. Often used as maintenance lock following infection control
Echinocandins (Caspofungin/Micafungin)	Variable concentrations reported (e.g., caspofungin ≈3.33 mg/mL; micafungin 0.05–0.15 mg/mL)	Saline/D5W	Caspofungin shows limited stability and possible incompatibility with heparin; micafungin appears more stable, based on limited data	*Candida* and *Aspergillus* spp.*;* inhibition of beta−1,3−D−glucan synthesis destabilizes fungal cell wall and biofilm matrix	Evidence for Echinocandins use is limited to experimental models and isolated clinical reports. Concentration−dependent and paradoxical effects described *in vitro*. Best considered adjunctive to systemic antifungal therapy rather than standalone lock solution

#### 5.2.1 Ethanol locks: the “nuclear option”

Ethanol is arguably the most potent agent available for destroying biofilm. It is a broad-spectrum microbicidal agent that affects bacteria and fungi indiscriminately by denaturing proteins and lysing cell membranes. A retrospect Cohort study reported an impressive 95% overall salvage rate for CRBSIs using a 4-hour, 5-day 70% ethanol lock regimen; however, all three catheters specifically infected with fungi failed salvage and required removal (Sampathkumar et al., 2025). Unlike antibiotics, it is not subject to classical resistance mechanisms. Clinical data suggests high efficacy for ethanol locks (typically 70% concentration) in sterilizing catheters and preventing CRBSI recurrence (Balestrino et al., 2009; Weber et al., 2024). Visweswaran et al. (2022) successfully salvaged a Candida-infected catheter, but this required a more prolonged regimen of 5-hour dwells over 6 consecutive days (Visweswaran et al., 2022). It is particularly effective against organisms that are resistant to standard antifungals. The major drawback of ethanol is its chemical interaction with catheter polymers. While silicone is generally resistant to alcohol, polyurethane catheters—which are common in hemodialysis—can be degraded by high concentrations of ethanol (Crnich et al., 2005). This degradation can manifest as structural weakening, cracking, or the elution of polymer components into the bloodstream. Ethanol precipitates plasma proteins, which can lead to the formation of proteinaceous sludge or clots within the catheter lumen (Schilcher et al., 2013). This increases the risk of catheter occlusion, potentially rendering the “salvaged” catheter useless for dialysis. There is an ongoing debate regarding the optimal concentration. While 70% ethanol is the standard for sterilization, lower concentrations (e.g., 30%) have been explored to mitigate catheter damage (Akıncı et al., 2024). However, *in vitro* data suggests that lower concentrations may fail to inhibit certain robust biofilms, making the 70% concentration a necessary risk for fungal salvage in many cases.

#### 5.2.2 Taurolidine: the safety standard

Taurolidine is a derivative of the amino acid taurine with broad-spectrum antimicrobial activity. It acts by a chemical reaction with the cell walls of bacteria and fungi, preventing biofilm formation and destroying existing cells (Betjes and van Agteren, 2004). Extensive systematic reviews and meta-analyses have shown taurolidine (often combined with citrate or heparin) to be highly effective in preventing CRBSIs, reducing the risk by approximately 50% compared to heparin alone (Vernon-Roberts et al., 2022; Wang and Sun, 2022). It has demonstrated fungicidal activity *against C. albicans* and *C. glabrata in vitro*. Unlike ethanol, taurolidine has an excellent safety profile. It does not degrade catheter materials and has no known systemic toxicity (Nguyen et al., 2024). It is arguably the “new standard” for lock prophylaxis in high-risk patients. However, while Taurolidine is the standard for lock prophylaxis, its strongest evidence remains in preventive use, and it has significant limitations regarding the eradication of an already established, mature fungal biofilm.

#### 5.2.3 Amphotericin B: the targeted approach

Amphotericin B (AmB) is a polyene antifungal that binds to ergosterol, forming pores in the fungal membrane and causing cell death. While AmB deoxycholate is the traditional formulation used for locks due to cost, lipid formulations (Liposomal AmB or AmB Lipid Complex) have shown unique efficacy in penetrating biofilms in animal models (Walraven and Lee, 2013; Imbert and Rammaert, 2018). A critical logistical challenge is compatibility with anticoagulants. AmB is incompatible with saline (it precipitates) and can precipitate with heparin if not prepared carefully. Protocols typically require the use of 5% Dextrose (D5W) as the diluent. Studies suggest that AmB (2.5 mg/mL) can be visually compatible with heparin (up to 5000 U/mL) for limited durations, but precipitation risks remain a significant concern that requires strict adherence to preparation protocols (Bailey et al., 2002; Bookstaver et al., 2013). Common protocols use concentrations of 2.0 - 2.5 mg/mL with dwell times matched to the inter-dialytic period (48–72 hours) (Blair et al., 2024).

### 5.3 The decision framework: when is salvage acceptable?

Synthesizing the available evidence, we propose a rigorous, risk-stratified decision framework for considering fungal catheter salvage. This approach emphasizes that salvage is an exception, not the rule. It is crucial to emphasize the evidence boundaries in this domain. Large-scale, head-to-head randomized controlled trials are currently lacking, and recommendations rely heavily on observational data and expert opinion. Therefore, catheter salvage must be viewed as a high-risk, exceptional “bridge” strategy rather than a standard or guaranteed cure.

Absolute Contraindications to Salvage: Clinicians must immediately proceed to catheter removal if the patient exhibits septic shock or hemodynamic instability. Evidence of metastatic infection, such as endocarditis, endophthalmitis, or osteomyelitis, mandates removal to control the source. Suppurative thrombophlebitis or a tunnel infection (erythema/purulence along the subcutaneous tract) are also absolute indications for removal, as extraluminal infections cannot be treated with intraluminal locks. Finally, if blood cultures remain persistently positive (>72 hours) despite appropriate systemic therapy, the catheter must be removed.

**Figure 3:**
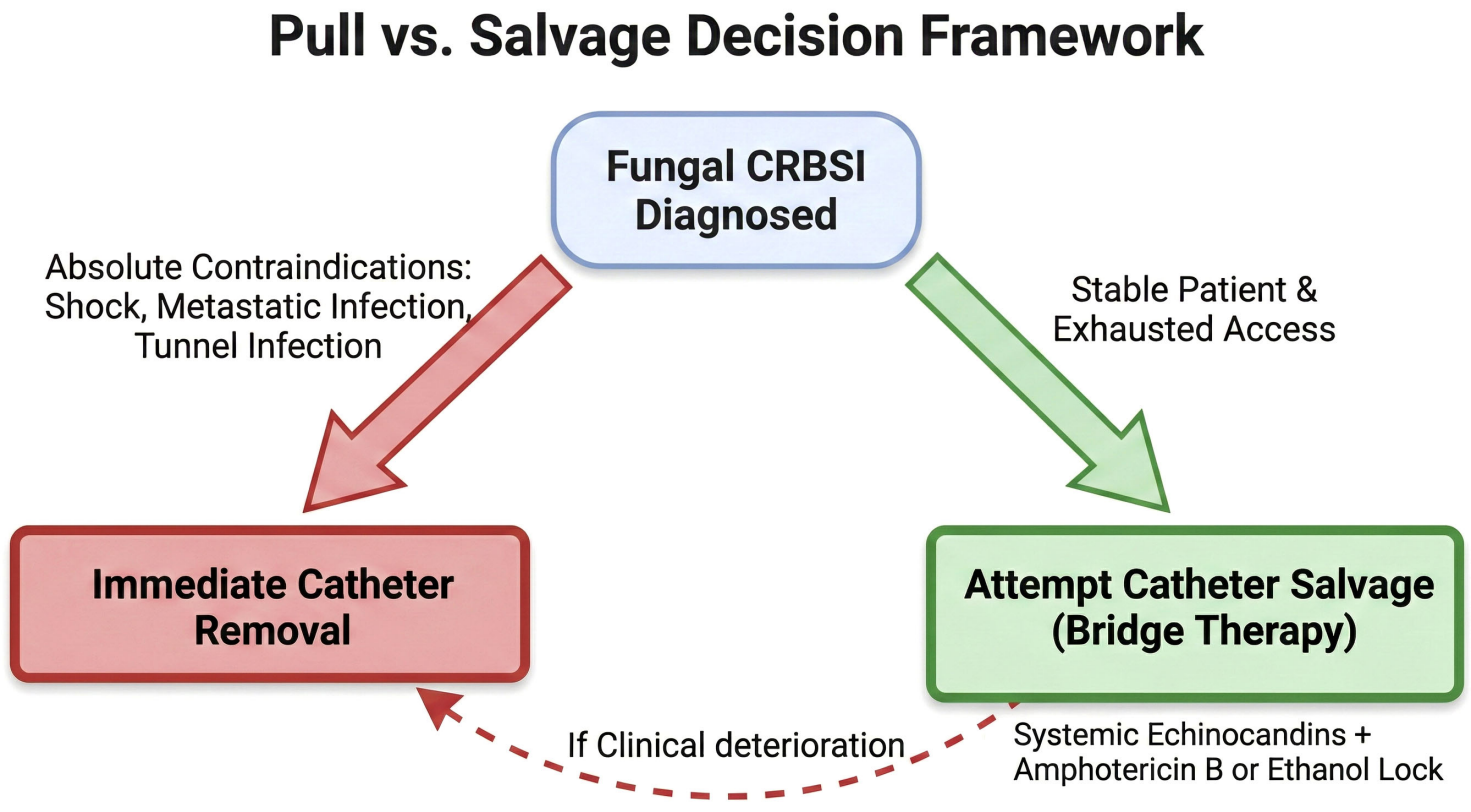
Clinical decision framework for the management of fungal catheter-related bloodstream infections (CRBSIs). Immediate Removal Pathway: Catheter retention is strictly contraindicated in the presence of septic shock, hemodynamic instability, metastatic infection (e.g., endocarditis, osteomyelitis), suppurative thrombophlebitis, tunnel infection, or if blood cultures remain persistently positive for >72 hours despite systemic therapy. Isolation of molds (e.g., Aspergillus) also mandates immediate removal. Attempted Salvage Pathway: Salvage should only be considered as a high-risk “bridge” therapy for hemodynamically stable patients with “exhausted access” (no viable alternative sites for recannulation or peritoneal dialysis) and a confirmed Candida species infection. Salvage Regimen and Monitoring: If salvage is attempted, it requires aggressive localized intervention with Antifungal Lock Therapy (e.g., Amphotericin B or Ethanol) combined with systemic echinocandins. Clinicians must maintain a very low threshold to abandon salvage and remove the catheter if the patient clinically deteriorates or fails to clear the fungemia.

Criteria for Attempted Salvage (The “Bridge”): Salvage may be considered only if the patient meets the criteria for “Exhausted Access,” meaning there are no other viable sites for immediate recannulation or the patient has a “hostile abdomen.” (peritoneal dialysis not possible) The patient must be hemodynamically stable. The pathogen should ideally be a *Candida* species; isolation of Aspergillus or other molds typically mandates immediate removal due to extremely high mortality. The goal of salvage in this context is often a “bridge” to a new access (e.g., stabilizing the patient to place a graft or fistula) rather than indefinite retention of the infected line. In this narrow window, the combination of Systemic Echinocandins + Amphotericin B (or Ethanol) Lock Therapy is theoretically positioned to offer the highest potential for clearance given the biofilm biology described. While large-scale head-to-head trials comparing lock regimens are lacking. The clinician must maintain a low threshold for abandoning salvage if the patient deteriorates or cultures remain positive. (**Figure 3**).

### 5.4 Guidewire-directed catheter exchange

As an alternative salvage strategy to preserve the vascular access site, guidewire-directed catheter exchange may be considered, a technique well-supported in the literature for managing catheter-related bacteremia without tunnel involvement (Beathard, 1999). This technique involves utilizing stiff wires threaded through both lumens, followed by the dissection and relief of the subcutaneous cuff. To mitigate the risk of dragging existing biofilm into the new catheter, a described procedural modification involves the rigorous 70% ethanol-based decontamination of the exposed part of the guidewires before the new, fresh catheter is threaded.

## 6 Prevention and stewardship

Given the difficulty of treating established fungal biofilms and the risks associated with salvage, prevention is the most effective strategy.

Prophylactic Locks: For high-risk patients—defined as those with a history of multiple CRBSIs, femoral catheters, or significant immunosuppression—the routine use of antimicrobial locks is supported by evidence. Taurolidine-Citrate locks have emerged as a superior alternative to standard heparin locks. Meta-analyses consistently indicate that taurolidine significantly reduces the overall incidence of CRBSIs compared to heparin (Liu et al., 2013, Liu et al., 2014). While clinical trials are often underpowered to detect statistically significant reductions specifically in fungal subgroups due to low event rates, *in vitro* data confirms taurolidine’s potent fungicidal activity against *Candida* species, supporting its use as a broad-spectrum preventive agent (Jafarian et al., 2022).

Stewardship: Antimicrobial stewardship is also critical. The overuse of broad-spectrum antibiotics selects for *Candida* overgrowth and predisposes patients to fungal infections. Minimizing the use of intradialytic parenteral nutrition (IDPN) through CVCs is also a crucial measure, as the lipid-rich and glucose-rich TPN solutions provide an ideal growth medium for *Candida* biofilms, particularly *C. parapsilosis*.

## 7 Conclusion

The management of fungal biofilms in hemodialysis catheters is a battle against a biologically sophisticated adversary. The fungal biofilm, with its complex architecture, ECM shielding, and persister cells, renders standard therapeutic approaches inadequate. While the “remove and replace” dogma of the IDSA and KDIGO guidelines remains the safest path for the majority of patients, it fails to address the desperate reality of the access-exhausted patient.

For this specific subgroup, catheter salvage is a high-stakes clinical gamble that requires expert execution. It demands a shift from passive systemic treatment to aggressive, localized intervention using ALT. The evidence supports the use of high-concentration agents like Amphotericin B or Ethanol (with caveats regarding catheter integrity) combined with systemic echinocandins to penetrate the biofilm matrix. However, this must be viewed as a bridging strategy rather than a guaranteed cure, given the high risk of relapse.

Ultimately, the future of nephrology lies in mastering these salvage techniques while simultaneously adopting robust preventive strategies, such as Taurolidine locking, to stop the “fungal stronghold” from being established in the first place. The gap between guideline and reality is bridged not by ignoring the rules, but by understanding the underlying biology deeply enough to know when and how to break them safely.
